# Agro-Industrial Plant Biomass as a Sustainable Source of Anticancer Polyphenols: Molecular Mechanisms and Future Perspectives

**DOI:** 10.3390/cimb48050459

**Published:** 2026-04-29

**Authors:** Sorur Yazdanpanah, Fabrizia Sepe, Silvia Romano, Anna Valentino, Orsolina Petillo, Gianfranco Peluso, Raffaele Conte, Anna Calarco

**Affiliations:** 1 Research Institute on Terrestrial Ecosystems (IRET)-CNR, Via Pietro Castellino 111, 80131 Naples, Italy; soruryazdanpanah@cnr.it (S.Y.); fabriziasepe@cnr.it (F.S.); silvia.romano@iret.cnr.it (S.R.); anna.valentino@cnr.it (A.V.); orsolina.petillo@cnr.it (O.P.); gianfranco.peluso@unicamillus.org (G.P.); anna.calarco@cnr.it (A.C.); 2 Department of Experimental Medicine, University of Campania “Luigi Vanvitelli”, Via Santa Maria di Costantinopoli 16, 80138 Naples, Italy; 3 National Biodiversity Future Center (NBFC), 90133 Palermo, Italy; 4 Faculty of Medicine and Surgery, Saint Camillus International University of Health Sciences, Via di Sant’Alessandro 8, 00131 Rome, Italy

**Keywords:** agro-industrial waste valorization, plant-derived polyphenols, anticancer bioactive compounds, circular economy, phytochemicals, chemoprevention, sustainable drug discovery

## Abstract

The increasing global burden of cancer, together with the need for more sustainable resource management, has stimulated growing interest in the valorization of agro-industrial plant residues as sources of bioactive compounds with therapeutic potential. This review highlights the potential of plant by-products—including citrus peels, olive leaves, date palm residues, and tea and coffee processing wastes—as sustainable reservoirs of polyphenols and other phytochemicals with significant anticancer activity. Key compounds such as hesperidin and naringenin from citrus peels, oleuropein and hydroxytyrosol from olive leaves, quercetin and syringic acid from date palm residues, and chlorogenic acid and epigallocatechin gallate from tea and coffee by-products have demonstrated promising antitumor effects in both in vitro and in vivo studies. These molecules exert their activity through multiple mechanisms, including the inhibition of cancer cell proliferation, induction of apoptosis, regulation of the cell cycle, and modulation of major oncogenic signaling pathways such as PI3K/AKT, MAPK, NF-κB, and EGFR. For instance, hydroxytyrosol induces apoptosis and cell cycle arrest while inhibiting the PI3K/AKT and MAPK pathways. Quercetin limits metastasis and glycolysis and suppresses VEGF, PKM2, and AKT signaling. Ferulic acid suppresses tumor growth by inhibiting the PI3K/AKT and JAK2/STAT6 pathways, thereby promoting apoptosis (in vitro and in vivo). In addition to their pharmacological potential, the recovery of these compounds from plant waste supports circular economy strategies by reducing environmental impact and promoting the development of value-added products. Future research should focus on optimizing extraction methods, improving bioavailability and stability, and validating safety and efficacy through well-designed preclinical and clinical studies.

## 1. Introduction

Global food and energy production systems generate vast quantities of agro-industrial residues [1]. Current estimates indicate that approximately 13.2% of total food production—equivalent to nearly 1.25 billion tons—is lost between harvest and the retail stage. In addition, more than 1 billion tons of food, representing around 19% of total global production, was wasted at the household, food service, and retail levels in 2022 [2]. This phenomenon results in substantial food waste, which contributes to greenhouse gas emissions, accelerates climate change, and poses serious challenges to global food security [3]. Consequently, food loss and waste represent a critical global issue with profound implications for environmental sustainability, food safety, and the capacity to nourish a growing population. However, strategies aimed at reducing food waste offer multiple benefits, including enhanced food security and nutrition, economic gains for stakeholders across the supply chain, and the mitigation of environmental impacts through reduced pollution and improved biodiversity conservation [4]. Addressing these issues requires technological innovation in recycling, waste treatment, and circular economy practices, all of which depend on adequate financial support. In this context, green finance plays a key role by channeling capital toward sustainable waste management projects, such as waste-to-energy and recycling systems. However, the uneven distribution of green financial resources may limit the diffusion of advanced waste technologies, reinforcing regional disparities. The effectiveness of green finance in tackling waste-related challenges is further enhanced when supported by strong financial capacity and active markets [5].

**Figure 1:**
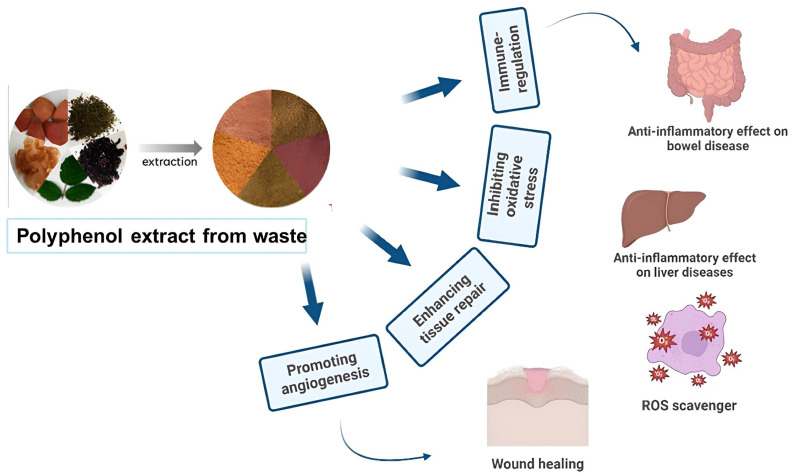
Phenolic compounds exert anticancer effects by inhibiting angiogenesis and metastasis (via VEGF and EMT modulation), suppressing proliferative pathways (e.g., PI3K/AKT, MAPK, STAT), inducing cell-cycle arrest, reducing oxidative stress, and promoting apoptosis through p53 activation and mitochondrial pathways. They also inhibit inflammation by suppressing NF-κB and pro-inflammatory mediators.

Among all of the food categories, fruit and vegetable waste represents one of the largest fractions, with considerably higher waste levels than cereals and pulses. This disparity is primarily attributed to the high perishability and short shelf life of fruits and vegetables. A large amount of agro-industrial plant by-products generated during processing—such as leaves, seeds, pomace, bran, peels, skins, oilseed meals, and other residual materials—highlight the urgent need for efficient management, recycling, valorization, or environmentally sound disposal practices [6]. In this context, nutraceuticals—defined as bioactive compounds derived from food sources and formulated into pharmaceutical-like products—have attracted increasing interest because of their health-promoting properties and protective effects against chronic diseases. Agro-industrial by-products, in particular, represent a valuable and underutilized source of such compounds, supporting the rapid growth of the nutraceutical sector [7]. Bioactive compounds, commonly referred to as secondary metabolites, are synthesized through plant metabolic pathways and exhibit significant potential health benefits, including antioxidants, antimicrobial, anti-inflammatory, and anticancer activities [8]. Metabolites are broadly categorized into primary and secondary types: primary metabolites, such as carbohydrates, proteins, lipids, and amino acids, are essential for basic cellular functions, whereas secondary metabolites are not directly involved in growth but play key roles in plant defense, adaptation, and ecological interactions. These compounds, which are often species-specific and influenced by environmental conditions, include biologically active molecules with significant therapeutic potential (Figure 1) [9]. Major classes of plant-derived bioactive compounds include phenolics, alkaloids, terpenoids, and glucosinolates [9]. Extensive research has investigated the anticancer properties of natural bioactive compounds across a wide range of malignancies, including breast [10,11], prostate [12,13], lung [14], liver [15], ovarian [16], pancreatic [17], and colorectal cancer [18]. It should be noted that chronic HBV infection is a major driver of hepatocellular carcinoma through viral mutations and alterations in tumor suppressor genes. Therefore, it is important to consider whether plant-derived compounds can mitigate these mechanisms. For instance, HBV mutations such as rtA181T are associated with increased HCC risk and genomic instability. Future studies should investigate whether phytochemicals can inhibit viral replication, reduce mutation burden, or protect tumor suppressor pathways [19]. Xanthohumol, a prenylated flavonoid from hops, exhibits broad anticancer activity by inhibiting proliferation and inducing apoptosis across multiple cancer types. Its effects are mediated through key pathways such as Akt, NF-κB, and ROS signaling, and it can enhance chemosensitivity in resistant cells. Despite its low toxicity, clinical application is limited by poor bioavailability [20]. These findings suggest that such compounds may effectively inhibit tumor initiation and progression. Regarding polyphenols, these compounds modulate the tumor immune microenvironment (TIME) by suppressing pro-inflammatory signaling pathways (e.g., NF-κB and STAT3), reducing the recruitment of immunosuppressive cells such as regulatory T cells (Tregs) and tumor-associated macrophages (TAMs), and enhancing antitumor immune responses through the activation of cytotoxic T lymphocytes and natural killer (NK) cells. Additionally, they can regulate immune checkpoint expression (e.g., PD-1/PD-L1) and decrease oxidative stress, thereby shifting the TIME from an immunosuppressive to an immunostimulatory state [21].

Importantly, many of these bioactive molecules can be recovered from plant-based agro-industrial by-products, reinforcing their dual role as both sustainable resources and promising anticancer agents. Notably, they may also act as chemosensitizers, enhancing the efficacy and reducing the toxicity of conventional chemotherapeutic drugs. However, despite their potential, significant economic bottlenecks remain, particularly related to the high costs of extraction, purification, and large-scale standardization, which may limit their industrial and clinical translation. In this context, the present review focuses on the anticancer potential of selected phenolic compounds obtained from upcycled plant biomass, highlighting their relevance within a circular economy framework.

**Figure 2:**
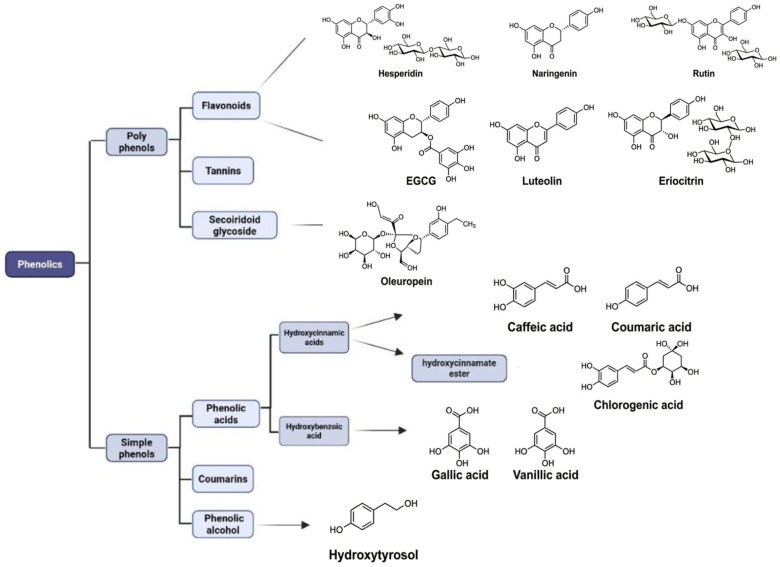
Classification of phenolic compounds and chemical structure of selected phenolics that may have anti-cancer potential.

In recent years, there has been growing interest in the efficient utilization of agricultural and food by-products as a strategy to enhance sustainability, reduce waste, and generate value-added products [22]. This growing interest is driven by several factors, including increased environmental awareness, advances in processing and extraction technologies, and the implementation of supportive regulatory policies. Sustainability, in this context, is centered on preserving natural resources to ensure their availability for future generations. In line with this objective, the concept of the circular economy has emerged as a viable alternative to the conventional linear “take–make–dispose” model [23,24]. The circular economy promotes efficient resource use through strategies such as reuse, repair, recycling, and remanufacturing, thereby extending the life cycle of materials and minimizing waste generation [25]. As a result, it contributes to reduced reliance on virgin resources and limits environmental pollution and carbon emissions [26]. Furthermore, Regulation (EC) 2008/98 on waste establishes a legal framework for waste management that promotes waste prevention, reuse, and recycling, encouraging the valorization of food by-products as raw materials rather than discards [27]. This directive clearly distinguishes food waste from by-products. Food waste refers to edible or inedible materials that are no longer suitable for consumption or use and are typically discarded without further valorization, such as leftovers, spoiled food, or unused ingredients. In contrast, by-products are secondary materials unintentionally generated during food processing or manufacturing that retain nutritional, functional, or economic value and can be repurposed for various applications [28,29]. The distinction between “waste” and “by-products” is crucial for valorization strategies, as it determines legal status, processing requirements, and economic feasibility within circular bioeconomy models. Indeed, based on this definition, the valorization of plant-derived by-products aligns with the core principles of the circular economy, as it enables the transformation of large volumes of materials traditionally considered waste into valuable raw resources. This approach not only reduces the environmental impact but also creates economic value. In particular, plant by-products have attracted increasing attention due to their rich content of bioactive compounds and are being extensively studied for their anticancer, anti-inflammatory, antioxidant, and antimicrobial properties, particularly in pharmaceutical applications [30]. Moreover, such compounds are widely utilized in the cosmetic industry for the formulation of skincare products [30], as well as in the food industry for the development of functional foods and nutraceuticals aimed at promoting health and preventing disease [7,31]. The integration of circular and green economy concepts has therefore contributed to a rapid expansion of research in this area. This trend is reflected in the growing number of scientific publications indexed in PubMed under topics related to food by-products and the circular economy, highlighting the increasing academic and industrial interest in this field [32,33]. Among the plant-derived bioactive compounds, phenolic compounds (Figure 2) represent one of the most abundant and extensively studied classes, particularly for their potential role in cancer prevention and therapy. Agro-industrial plant by-products constitute an important and largely underutilized source of these molecules. Chemically, phenolic compounds are characterized by the presence of one or more hydroxyl (–OH) groups attached to aromatic rings, resulting in a broad structural diversity that ranges from simple phenolic acids to more complex polyphenols such as flavonoids and high-molecular-weight tannins. These compounds are generally classified into three main groups: flavonoids, phenolic acids, and tannins [34]. Flavonoids represent one of the most abundant and structurally diverse subclasses of polyphenols. Their basic structure consists of a 15-carbon skeleton arranged in a C6–C3–C6 configuration, comprising two benzene rings (A and B) linked by a three-carbon bridge that typically forms a heterocyclic pyran ring (C). Structural variations within this backbone, particularly in the degree of hydroxylation, methoxylation, and glycosylation, are used to classify flavonoids into different subclasses, including flavones, flavonols, isoflavones, and flavanones. Flavanones, such as naringenin and hesperidin, are characterized by a saturated C ring, lacking the double bond between C2 and C3 that is present in other flavonoid subclasses. As secondary metabolites produced during plant biosynthesis, flavonoids contain multiple phenolic (aromatic hydroxyl) groups that are primarily responsible for their biological activities [34]. Among the flavanones, hesperidin is a well-studied bioflavonoid exhibiting a wide range of pharmacological properties, including antioxidant, anti-inflammatory, cardioprotective, neuroprotective, antimicrobial, and anticancer effects. Its anticancer potential has been extensively investigated in preclinical studies, where it has been shown to modulate key molecular pathways involved in cell proliferation, apoptosis, and tumor progression. The biological activity of hesperidin is largely attributed to the presence and position of hydroxyl groups on both the aromatic rings and the heterocyclic C ring, which influence its ability to interact with cellular targets and regulate oxidative and signaling processes. Phenolic compounds are widely recognized for their strong antioxidant activity, largely attributed to their ability to neutralize free radicals and reactive oxygen species. While early research primarily focused on these antioxidant properties to explain their health benefits, more recent studies have demonstrated that their biological effects are more complex. In particular, phenolic compounds can modulate key cellular signaling pathways, including those involving protein kinases and lipid-mediated signaling mechanisms [35]. Beyond their antioxidant capacity, they exhibit a wide spectrum of biological activities, including anticancer, anti-inflammatory, and antimicrobial effects [36]. The following section therefore focuses on the anticancer potential of phenolic compounds derived from selected plant by-products, with particular emphasis on the molecular mechanisms underlying their biological activity.

## 2. Phenolic Compounds Recovered from Plant by Products

### 2.1. Citrus Peels

Citrus fruits are among the most extensively cultivated fruit crops globally, with a reported worldwide production of 169 million metric tons in 2023 [37]. Among the cultivated citrus species, oranges (*Citrus sinensis*) represent the predominant crop, followed by mandarins and related species (*C. reticulata*, *C. unshiu*, and *C. tangerina*), as well as grapefruits (*C. paradisi*), lemons (*C. limon*) and limes (*C. aurantifolia*), among others [38]. During citrus processing, a large amount of organic waste is generated, including seeds, peels, and residues remaining after juice extraction. Among these by-products, citrus peels constitute the largest proportion of citrus waste, accounting for approximately 50–55% of the total fruit waste [39,40]. Citrus peel is a low-priced and readily available by-product that can be effectively utilized as a valuable source of bioactive compounds in the pharmaceutical, nutraceutical, and food industries. In this context, it is important to note that the composition and bioactivity of phenolic compounds in citrus peels may vary depending on processing methods such as fermentation. These processes can induce transformations, including the release, modification, and biosynthesis of phenolics, which may influence their functional properties. Considering these aspects in future studies may provide a more comprehensive understanding [41].

Numerous studies have highlighted the nutritional and health-promoting properties of citrus fruits, demonstrating a broad spectrum of biological activities, including anticancer, antidiabetic, antiplatelet, anti-inflammatory, and antimicrobial effects [42]. In this regard, Eman A Abdelghffar et al. recently demonstrated that orange peel extract exerts a significant protective effect against organ toxicity induced by cyclophosphamide, a commonly used anticancer and immunosuppressive drug, in an in vivo model. It was demonstrated that orange peel extract may exert a significant prophylactic effect against cyclophosphamide-induced hepatic, renal, and cardiac injury by attenuating inflammation (serum TNF-α, IL-1β, and IL-6) and reducing lipid peroxidation. Moreover, increased antioxidant markers—including serum total antioxidant capacity (TAO) and tissue levels of glutathione (GSH) and catalase (CAT)—further supported the protective efficacy of orange peel extract against cyclophosphamide toxicity [43]. Flavonoids, a family of natural polyphenolic compounds, are commonly found in fruits and vegetables, including citrus. In citrus waste, flavonoids are mainly located in the peel, pulp, and rag tissues. Major flavonoids identified in fruits of citrus origin include naringin, hesperidin, eriocitrin, and narirutin [44]. Extensive evidence indicates that citrus-derived flavonoids possess significant anticancer properties [45], with breast cancer being the most intensively investigated model [46,47,48,49,50,51]. In this context, naringenin, one of the major flavonoids in citrus fruits, has been reported to suppress postoperative metastatic growth in breast cancer through the modulation of host immune responses [52]. Beyond breast cancer, the anticancer potential of citrus flavonoids has been documented in a broad spectrum of tumors, including colorectal [53,54,55,56], gastric [57,58], hepatic [59,60], pulmonary [61,62,63], prostate [64,65], cervical [66,67], ovarian [68,69], and epidermal cancers [70,71]. Mechanistically, citrus flavonoids exert their therapeutic effects by interfering with key processes involved in tumor progression. These include the suppression of cancer cell proliferation and migration [72], inhibition of angiogenesis, and activation of programmed cell death pathways [73]. Supporting this, hesperidin has been found to significantly reduce cell growth and induce apoptotic signaling in non-small cell lung cancer cell lines, including A549 and NCI-H358 [74]. Similarly, naringenin has been shown to promote apoptosis while inhibiting proliferation, migration, and invasion in gastric carcinoma [75]. Consistent with these findings, in vivo studies have shown that hesperidin exhibits anticarcinogenic activity against colorectal cancer by inducing apoptosis and inhibiting cell proliferation [76,77]. It is important to distinguish between studies using crude extracts and those investigating purified compounds. A crude extract is a complex mixture obtained directly from plant material using solvents or other extraction techniques. It contains a wide range of constituents. The observed biological effects of crude extracts often result from the combined or synergistic interactions among multiple components, making it difficult to attribute the activity to a single molecule. In contrast, purified compounds are individual chemical entities that have been isolated and structurally characterized from the crude extract. Studying purified compounds allows for a more precise evaluation of their specific biological activity, mechanism of action, and dose–response relationship. Many reported biological effects of citrus-derived products are based on crude extracts, which contain complex mixtures of bioactive constituents and may exhibit synergistic or additive effects. In contrast, studies using purified compounds allow for more precise mechanistic insights but may not fully reflect the complexity of biological systems. Therefore, this distinction should be considered when interpreting the reported anticancer activities. In a very recent study, it was reported that lemon peel extract contains ten phenolic acids and eleven flavonoids, with ferulic acid and hesperidin as the most abundant constituents. Lemon peel extract altered the cellular redox dynamics. This alteration was characterized by reduced hydrogen peroxide-related ROS levels, increased superoxide anion accumulation, and decreased expression of antioxidant enzymes including superoxide dismutase and catalase, indicating a broad disruption of redox homeostasis that ultimately compromises cancer cell viability and survival. In addition to polyphenols, plant-derived wastes such as tiger nut and oilseeds may also serve as sources of bioactive peptides with antioxidant properties. These peptides may act synergistically with polyphenols, potentially enhancing their overall biological effects and contributing to a more comprehensive “waste-to-value” approach. This aspect may also be considered in future studies [78].

Accordingly, lemon peel extract induced dose- and time-dependent cytotoxic effects in gastric cancer cell lines (AGS and MKN-28), causing marked morphological alterations while sparing normal BJ-5ta fibroblasts [79]. Flavonoids isolated from citrus peel—quercetin, naringenin, and naringin—reduced the viability of estrogen-dependent breast cancer cells (MCF-7 and T47D) without affecting normal melanocytes, indicating a good safety profile. Since aromatase is the key enzyme responsible for estrogen production, and estrogen stimulates hormone-dependent breast cancer growth, these compounds were further tested in an in vivo tumor model. They significantly reduced the tumor volume and decreased the aromatase levels, suggesting that their anticancer effect is mediated through aromatase inhibition [80]. Another important citrus flavonoid, eriocitrin, has demonstrated notable anticancer properties. Eriocitrin derived from lemon suppresses the proliferation of human hepatocellular carcinoma cells by inducing apoptosis and causing cell cycle arrest. Eriocitrin, a flavonoid found in lemon and other citrus fruits, exhibits a wide range of biological activities, including antioxidant, antitumor, anti-inflammatory, and anti-allergic effects. It has been demonstrated to promote apoptosis through activation of the intrinsic (mitochondrial) pathway and may also involve modulation of the MAPK signaling pathway [81]. Eriocitrin exerts anti-angiogenic and anticancer effects primarily in HUVECs by inhibiting VEGFR2 phosphorylation, thereby suppressing key downstream pathways including PI3K/AKT/mTOR and MAPK/ERK, which regulate endothelial cell proliferation, survival, and migration. It also induces apoptosis via the intrinsic pathway through activation of caspase-9, caspase-3, and PARP cleavage, leading to endothelial cell death. Additionally, eriocitrin reduces extracellular matrix degradation and cell migration by downregulating MMP-2 and MMP-9. These effects were observed in vitro in HUVECs and supported by in vivo CAM models, as well as in cancer cell lines such as A549, H1299, HepG2, and Huh7, where it inhibits proliferation, migration, and promotes apoptosis. [82]. Table 1 recaps the molecular actions of polyphenols on tumor target processes, providing information on their structural features and structure–activity relationships.

### 2.2. Olive Leaves

Olive leaves are generated in large quantities as an agro-industrial by-product of olive tree cultivation and olive oil production. Significant amounts are produced annually during harvesting and routine pruning practices, which yield approximately 25 kg of residual biomass per olive tree, comprising branches and a substantial proportion of leaves [83]. Additional olive leaves are removed and discarded during the washing of olive drupes at the initial stage of the olive oil production process. Despite being traditionally considered agricultural waste, olive leaves represent a valuable plant by-product due to their high content of bioactive constituents [83]. Both olive-derived products and by-products are rich in biologically active compounds, with olive leaves being particularly abundant in phenolic substances. The main phenolic classes identified in olive leaves include (i) secoiridoids, such as oleuropein and its aglycone derivatives; (ii) simple phenols, notably hydroxytyrosol and tyrosol; and (iii) flavonoids, including rutin and luteolin-7-glucoside [84]. Among these compounds, oleuropein and hydroxytyrosol are present at especially high concentrations in olive leaves [85]. Hydroxytyrosol has been shown to exert dose-dependent effects, including the inhibition of proliferation through G1/S cell cycle arrest and the induction of apoptosis via caspase activation and PARP cleavage [86].

As a result, olive leaves constitute an inexpensive, renewable, and widely available source of phenolic compounds with well-documented health-promoting properties [87]. In particular, oleuropein and hydroxytyrosol have been extensively reported to exhibit anticancer activity, both as isolated compounds and as adjuvants in pharmaceutical applications. These phenolics have demonstrated in vitro antitumor effects across a broad range of cancer cell models, including breast cancer, melanoma, cervical, ovarian, colon and colorectal adenocarcinoma, hepatocellular carcinoma, osteosarcoma, head and neck squamous cell carcinoma, neuroblastoma, as well as hematological malignancies such as leukemias, myelomas, and lymphomas [88]. Moreover, preliminary findings showed that oleuropein time-dependently inhibited MCF-7 cell proliferation [89]. Mechanistically, oleuropein induced apoptosis in MDA-MB-231 triple-negative breast cancer cells by activation of NF-κB signaling. This effect was associated with increased ROS accumulation and inhibition of the NF-κB signaling cascade [90]. Specifically, oleuropein inhibits the NF-κB signaling cascade, leading to reduced transcription of downstream pro-survival and anti-apoptotic genes, thereby contributing to apoptosis induction in MDA-MB-231 cells [90]. In another study conducted using the same cell models, oleuropein, both in its isolated form and as part of olive leaf extract, significantly reduced cell proliferation and induced apoptosis in hormone receptor-positive (MCF-7) and triple-negative (MDA-MB-231) breast cancer cell lines at low concentrations. In addition to its cytotoxic effects, oleuropein impaired cell motility and reduced cellular spreading, probably by interfering with cytoskeletal remodeling, thereby suggesting a potential role in suppressing metastatic behavior [91]. Another study showed that oleuropein exerted cytotoxic and antiproliferative effects associated with S-phase cell cycle arrest and apoptosis induction. This response was accompanied by marked upregulation of apoptosis-related genes, including pro-apoptotic regulators (BNIP2, BNIP3, BID, and BCL10), components of the death receptor signaling pathway (FADD and TNFRSF21), as well as CYCS, CFLAR, and GADD45A, indicating the activation of multiple apoptotic signaling pathways [92]. In head and neck cancer cells, oleuropein significantly decreased cell viability and induced apoptosis via upregulation of the pro-apoptotic protein BAX and the tumor suppressor PTEN while concomitantly downregulating CDK2 and CDK4, which are key regulators of cell cycle progression and essential for cellular proliferation, thereby inducing cell cycle arrest [93]. Additional findings showed that oleuropein markedly reduced the viability of oral squamous cell carcinoma cells by promoting β-TRCP-mediated ubiquitination and degradation of Mcl-1, a key anti-apoptotic protein, through inhibition of the Akt–GSK3β–Mcl-1 signaling pathway. Consistent with these in vitro results, oleuropein significantly delayed tumor growth in vivo without inducing toxicity in major organs, highlighting its potential as a promising antitumor agent for oral squamous cell carcinoma cells [94]. A very recent investigation demonstrated that oleuropein induced DNA damage in prostate cancer cells, leading to reduced cell viability and increased apoptotic cell death in the DU145 prostate cancer cell line. These effects were associated with the activation of DNA damage response pathways and were suggested to be mediated, at least in part, through modulation of the HIF-1α signaling pathway [95]. Hydroxytyrosol, a key bioactive compound found in olive leaves, exerts multifaceted anticancer effects by targeting key cellular processes involved in cancer development and progression [96,97,98]. Acting as a potent antioxidant under physiological conditions, hydroxytyrosol reduces reactive oxygen species and oxidative stress, thereby supporting cellular homeostasis and contributing to cancer chemoprevention by limiting oxidative DNA damage and tumor initiation [99]. Hydroxytyrosol also modulates inflammatory pathways implicated in cancer progression, suppressing pro-tumorigenic signaling within the tumor microenvironment. In addition, it regulates cell cycle progression by inducing cell cycle arrest and promoting apoptosis [100,101,102]. Furthermore, hydroxytyrosol interferes with critical signaling pathways, including PI3K/AKT and MAPK, thereby inhibiting cancer cell survival, growth, and metastatic potential [103]. In addition, hydroxytyrosol exhibited anticancer activity in the acute human leukemia cell lines Jurkat and HL-60 by inducing G0/G1 cell cycle arrest and promoting apoptosis. These antiproliferative effects were associated with inhibition of the PI3K signaling pathway [104]. In a recent investigation, Aghaei et al. reported that hydroxytyrosol induced apoptosis in breast cancer cell lines (MDA-MB-231 and MCF-7) through the increased expression of the pro-apoptotic genes BAX and caspase 3 and reduced expression of the anti-apoptotic gene BCL2 [105]. Hydroxytyrosol promotes apoptotic cell death in LS180 colorectal cancer cells through the upregulation of key pro-apoptotic mediators, including BAX, caspase 3, and p53, along with an elevated BAX/BCL-2 ratio. Concurrently, it suppresses NFE2L2 expression, thereby weakening antioxidant defense mechanisms in cancer cells and facilitating apoptosis [106]. Therefore, olive leaves represent an abundant and low-cost natural resource rich in bioactive compounds with significant pharmacological potential, as described in Table 2.

### 2.3. Date Palm Residues

Date palm fruits are widely available in global markets and are consumed extensively across diverse regions of the world, particularly in the Middle East and Arabian countries [107]. The widespread consumption of this food is accompanied by the production of large volumes of processing waste, arising from preharvest losses and inefficient postharvest handling [108]. Date palm seeds alone account for approximately one million tons of by-products annually, posing significant environmental and economic challenges when managed through conventional disposal methods [109]. In line with circular economy principles, these by-products represent a rich and underutilized source of valuable bioactive and nutritional compounds. Their abundance and favorable composition make low-grade date fruits, pomace, and seeds promising raw materials for the production of value-added products in the food, pharmaceutical, nutraceutical, cosmetic, agricultural, and bio-based industries. Different studies have demonstrated substantial variability in the phenolic composition of dates, depending on cultivar and fruit part. In date flesh, several phenolic acids and flavonoids have been reported, including gallic, caffeic, chlorogenic, vanillic, cinnamic, ferulic, and coumaric acids, as well as catechins, rutin, kaempferol, quercetin, luteolin, and related compounds [110,111]. In contrast, date seed extracts generally exhibit a richer and more diverse phenolic profile, with the frequent identification of catechins, epicatechin, gallic acid, caffeic acid, chlorogenic acid, coumaric acid, sinapic acid, vanillic acid, rutin, quercetin, and luteolin, among others [111]. Indeed, a study by Habib et al. demonstrated the multifaceted therapeutic potential of date seed extract, highlighting its potent antioxidant and metal-chelating activities. These effects are attributed to free radical scavenging and the chelation of labile iron, which prevent iron-induced DNA and protein damage and thereby protect against oxidative stress. In addition, date seed extract inhibited acetylcholinesterase, α-amylase, and tyrosinase, enzymes implicated in several prevalent diseases, and exerted dose-dependent antiproliferative effects on hepatic, colorectal, and breast cancer cells through apoptosis, accompanied by the downregulation of BCL-2 and upregulation of p53 expression [112]. In another study, the antitumorigenic effects of date palm pit extracts from different Emirati varieties were evaluated in triple-negative breast cancer cells. Pit extracts demonstrated significant cytotoxicity toward MDA-MB-231 cells and induced marked morphological changes. Phosphokinase analysis suggested that these effects are mediated through the EGFR/ERK/FAK signaling pathway, which regulates cancer cell proliferation, survival, and migration, as well as Src family kinases, key drivers of oncogenic signaling and tumor invasiveness. Additionally, modulation of calcium signaling, a critical regulator of apoptosis and cell cycle control, was observed, indicating a coordinated disruption of pathways essential for tumor cell viability [113]. Another investigation evaluated the pharmacological effects of date pit extract in three pancreatic cancer cell lines and demonstrated significant antiproliferative and pro-apoptotic activity [114]. A further study reported that both leaf and seed extracts from the Khalas date palm cultivar exerted cytotoxic effects; notably, the leaf extract exhibited significantly greater antiproliferative activity than the seed and fruit extracts in prostate and pancreatic cancer cells, an effect associated with higher phenolic and flavonoid contents and enhanced antioxidant capacity. Moreover, the seed extract exerted its anticancer effects primarily through the induction of apoptosis, as evidenced by G2/M cell cycle arrest and the upregulation of apoptosis-related genes, including caspase-3 and caspase-9 [115]. In addition, Mouna Chakroun et al. also demonstrated the therapeutic potential of date palm leaf extracts from four cultivars, showing significant inhibition of tumor growth in MDA-MB-231 breast cancer and U87 glioblastoma cells. The extracts reduced cancer cell adhesion to fibrinogen and fibronectin by interfering with the αvβ3 and α5β1 integrin receptors and significantly suppressed cell migration, as confirmed by wound-healing assays [116]. Phenolic compounds present in date palm also possess therapeutic properties against colorectal cancer. For example, an in vivo study showed that the oral administration of syringic acid for 15 weeks significantly reduced the tumor growth and incidence in rats with DMH-induced colorectal cancer. These effects were primarily mediated through the inhibition of cell proliferation, induction of ROS-mediated apoptosis and DNA damage, and suppression of key genes involved in tumor progression [117]. One of the most extensively studied phenolics present in palm date pit and flesh extracts is quercetin. Both in vitro and in vivo studies have demonstrated that quercetin exerts potent antitumor effects by modulating cell cycle progression, inhibiting cell proliferation, inducing apoptosis, suppressing angiogenesis and metastasis, and regulating autophagy. Several in vivo investigations in prostate cancer models have shown that quercetin enhances apoptotic activity while reducing tumor cell proliferation through the downregulation of the androgen receptor (AR) and PI3K/AKT signaling pathways [118], as well as by inhibiting angiogenesis via the modulation of thrombospondin-1 (TSP-1) [119]. In breast cancer in vivo models, quercetin has been reported to induce apoptosis [120], inhibit metastasis and glycolysis, and suppress the expression of VEGF, PKM2, and the p-AKT/AKT signaling axis [121]. Furthermore, other studies have revealed that quercetin induces cell cycle arrest and suppresses the AKT/mTOR pathway [122] while also inhibiting angiogenesis through the downregulation of the calcineurin/NFAT signaling pathway [120]. In colorectal cancer models, quercetin reduces metastasis and induces apoptosis [123], whereas in hepatocellular carcinoma, it inhibits tumor growth [124]. More recently, using a triple-negative breast cancer (TNBC) model, the study showed that quercetin markedly reduced tumor growth and lung metastasis promoted by chronic stress. To investigate the underlying mechanisms, epinephrine was applied as a stress-related hormone in the in vitro assays. The findings revealed that quercetin suppressed the epinephrine-driven proliferation and migratory behavior of TNBC cells by inhibiting the β2-adrenergic receptor–ERK1/2 signaling cascade. Overall, these results indicate that quercetin interferes with stress-induced ERK1/2 activation in TNBC cells, thereby restraining tumor progression and metastatic dissemination [125]. Consistent with these observations, quercetin treatment triggered apoptotic cell death across all examined cancer cell lines at the tested doses. A marked increase in apoptosis was observed in mouse colon carcinoma (CT-26), human prostate adenocarcinoma (LNCaP), human T-cell acute lymphoblastic leukemia (MOLT-4), and human Burkitt’s lymphoma (Raji) cells. In vivo studies showed that quercetin administration significantly decreased the tumor volume in mice bearing MCF-7 and CT-26 xenografts [120]. Syringic acid exhibits strong antioxidant and anti-inflammatory properties through several molecular mechanisms. It alleviates oxidative stress by neutralizing free radicals, strengthening endogenous antioxidant defense systems, and activating the Nrf2 signaling pathway. In addition, syringic acid attenuates inflammatory responses by inhibiting the HMGB1/TLR4/MyD88/TRAF6/NF-κB signaling cascade. Interactions among the Nrf2, NF-κB, and PI3K/AKT pathways further indicate that syringic acid can regulate important cellular processes, including apoptosis, ferroptosis, and endoplasmic reticulum stress [126]. Another phenolic compound, ferulic acid, exhibits broad anticancer activity by targeting key oncogenic signaling pathways, particularly PI3K/AKT and JAK2/STAT6, which are frequently hyperactivated in cancer cells. Inhibition of these pathways suppresses tumor growth, limits tumor establishment, and induces programmed cell death, highlighting ferulic acid as a multi-target anticancer agent with a favorable safety profile [127]. Gallic acid exhibited significant anticancer effects on human embryonic carcinoma cells (NTERA-2 and NCCIT) by inducing apoptosis and G0/G1 cell cycle arrest through the modulation of key cell cycle regulators. It also reduced stem-like characteristics by downregulating cancer stem cell markers (SOX2, NANOG, and OCT4), triggered oxidative stress–mediated DNA damage responses, and inhibited invasion and migration via suppression of the EGFR/JAK2/STAT5 pathway [128]. A subsequent study showed that caffeic acid suppressed proliferation and migration in malignant pleural mesothelioma cells by reducing Ki67 and PCNA expression and inhibiting colony formation. These effects were mediated through the concentration-dependent inhibition of ERK1/2 and AKT signaling, key pathways regulating cell proliferation and survival. Caffeic acid also induced G2/M cell cycle arrest via the p53-dependent upregulation of p21 and p27 and triggered mitochondrial apoptosis, as indicated by an increased Bax/Bcl-2 ratio and caspase-3 activation [129]. The anticancer mechanisms of polyphenols derived from date palm residues are summarized in Table 3.

### 2.4. Tea and Coffee By-Products

Spent tea leaves and coffee grounds represent abundant agro-industrial wastes that are particularly rich in bioactive compounds such as epigallocatechin gallate (EGCG), chlorogenic acid, and caffeine. These molecules have been widely documented for their ability to inhibit cell cycle progression, induce apoptosis, and modulate oncogenic signaling pathways involved in cancer development and progression [130,131].

Coffee is one of the most widely consumed beverages worldwide, with global demand reaching approximately 173.1 million 60-kg bags during the 2022/2023 coffee year [132]. Given this high level of consumption, increasing attention has been directed toward the environmental impact associated with the large quantities of residues generated by the coffee industry. Consequently, numerous studies have focused on the valorization of coffee by-products within the framework of the circular economy [133]. Coffee by-products include a variety of materials generated at different stages of coffee production, ranging from cultivation to processing and final consumption. Among the most common by-products are spent coffee grounds, which remain after the brewing process; coffee husks, the protective outer layer (husk or parchment) that encloses the coffee beans and is removed during processing; coffee cherry pulp, which corresponds to the fruit surrounding the coffee bean and remains after the beans are separated from the cherries; and coffee silverskin, the thin silver-colored membrane that covers the coffee bean and is removed during the roasting process [134]. These by-products have attracted growing scientific interest because they contain high levels of bioactive compounds and nutrients with potential biological activity. In particular, phenolic compounds are abundant in these residues, with chlorogenic acid and its derivatives representing the predominant phenolic constituents [135]. Substantial research has demonstrated that coffee intake may exert chemopreventive and antineoplastic effects, particularly in relation to the development and progression of colorectal cancer [136]. The potential protective role of coffee has been attributed, at least in part, to its chlorogenic acid content, which is believed to confer anti-carcinogenic properties by reducing intestinal inflammation and mitigating oxidative stress within the epithelial lining of the gut [137]. In a related investigation, sixteen phenolic compounds were identified in coffee by-products using high-performance liquid chromatography coupled with electrospray ionization multi-stage mass spectrometry, with chlorogenic acids reported as the predominant constituents [138]. Chlorogenic acid exhibits anticancer activity by inducing cell cycle arrest, promoting apoptosis, and inhibiting tumor cell proliferation through the modulation of immune-related genes and induction of DNA damage. These findings highlight chlorogenic acid as a promising natural compound with potential anticancer activity [139]. Consistent with previous reports, chlorogenic acid, together with green and roasted coffee extracts, significantly inhibited HepG2 cell viability and proliferation in a dose-dependent manner, with minimal involvement of the Wnt/β-catenin signaling pathway, suggesting that the observed cytotoxic effects may involve apoptosis-related mechanisms [140]. For another, numerous in vitro and in vivo studies employing colorectal cancer models have demonstrated that treatment with coffee and its bioactive constituents can arrest the cell cycle at the G0/G1 phase, reduce cellular proliferation, and trigger apoptotic pathways [141]. Roasted coffee powder extracts have shown dose-dependent inhibitory effects on the growth of human colon Caco-2 cells. The underlying molecular mechanisms are thought to involve the upregulation of specific microRNAs, including miR-30c and miR-96, along with the suppression of KRAS proto-oncogene expression, thereby modulating key signal transduction pathways associated with tumor progression [142]. Further evidence comes from the study of Verrillo et al., in which the anticancer potential of coffee waste against colorectal cancer cells was evaluated, and chlorogenic acid was identified as the major bioactive component. In vitro findings demonstrated that coffee waste selectively inhibited HT-29 colorectal cancer cells’ viability, migration, and proliferation by inducing programmed cell death through activation of the TNF-α signaling pathway and disruption of calcium homeostasis. Additionally, its strong antioxidant activity suggests selective cytotoxicity toward cancer cells while sparing normal cells [135].

Tea production generates substantial agro-industrial waste at multiple stages of cultivation and processing, which, if improperly managed, can lead to environmental pollution. Sustainable management and valorization of these residues through biological and thermochemical conversion processes can reduce greenhouse gas emissions, enhance environmental sustainability, and support circular economic approaches [143]. Tea, derived from Camellia sinensis, is rich in biologically active constituents, including catechins and caffeine [144]. Typically, four major epicatechin-type catechins are identified in the fresh leaves of conventional tea cultivars: epicatechin (EC), epigallocatechin (EGC), epicatechin gallate (ECG), and epigallocatechin gallate (EGCG) [144]. It has been shown that EGCG is the predominant phenolic constituent of green tea [145], and EGCG and EC were successfully isolated from tea waste with minimal degradation during the extraction process [146]. EGCG’s broad-spectrum antitumor properties have been extensively demonstrated in both in vitro and in vivo studies. Research has shown that EGCG exerts anticancer effects across a wide range of malignancies derived from different tissues, including bladder, breast, cervical, colorectal, gastric, hepatic, lung, and head and neck cancers. For example, a recent study demonstrated that EGCG inhibits the EGFR signaling pathway in lung cancer cells. In A549 and HCC827 cells, EGFR inhibition was associated with the altered phosphorylation of mTOR, p38 MAPK, and STAT3. Moreover, EGCG induced apoptosis in non-small lung cancer cells (H1975). Overall, EGCG reduced cell migration and modulated the expression of vinculin and metavinculin—cytoskeletal proteins that regulate cell adhesion and migration—across non-small lung cancer cell lines [147]. In another investigation, EGCG, a bioactive compound derived from green tea, demonstrated significant inhibitory effects on tumor development and progression. In bladder cancer cell lines (5637 and T24), low concentrations of EGCG suppressed cell proliferation and promoted apoptosis, as evidenced by the increased expression of apoptosis-related proteins, including caspase-9, caspase-3, and BAX. Furthermore, EGCG regulated autophagy-related proteins (LC3B-II and Beclin) and its effects were reduced by PI3K/AKT inhibition and ATG5 silencing. ATG5 (Autophagy-Related Gene 5) is a key protein required for the formation of the autophagosome, suggesting that EGCG exerts anticancer activity partly through autophagy modulation [148]. EGCG-induced autophagy functioned in a pro-death (cytotoxic autophagy) context in the reported models. Inhibition of ATG5 attenuated EGCG-induced cytotoxicity, indicating that autophagy contributes to cell death rather than survival under these experimental conditions [148]. A further study comparatively assessed the anticancer effects of green tea extract and EGCG in a three-dimensional (3D) MCF-7 breast cancer model. Exposure of MCF-7 spheroids to green tea extract or EGCG demonstrated that the green tea extract more effectively suppressed spheroid formation and cell migration while inducing more pronounced morphological changes. These results suggest that the complex phytochemical composition of green tea extract may enhance its anticancer activity, possibly through synergistic interactions among its bioactive constituents [149]. Green tea extract and EGCG also demonstrated significant antitumor effects by inhibiting melanoma cell proliferation through apoptosis. Both compounds also exhibited anti-angiogenic activity and reduced TNF-α-induced VEGF and IL-8 secretion, supporting their potential role in cancer therapy [150]. Table 4 recaps the cited examples.

## 3. Conclusions

The increasing global burden of cancer, together with the urgent need for sustainable resource management, has stimulated growing interest in the valorization of agro-industrial plant residues as sources of bioactive compounds with therapeutic potential. As highlighted throughout this review, a wide range of plant-derived wastes—including citrus peels, olive leaves, date palm residues, and tea and coffee by-products—represent abundant and renewable sources of polyphenols and other phytochemicals with significant anticancer properties. These materials, traditionally regarded as low-value agricultural waste, are now recognized as reservoirs of biologically active molecules such as flavonoids, phenolic acids, and catechins. Compounds including hesperidin and naringenin from citrus peels, oleuropein and hydroxytyrosol from olive leaves, quercetin and syringic acid from date palm residues, and chlorogenic acid and epigallocatechin gallate from coffee and tea by-products have demonstrated remarkable antitumor activities in numerous in vitro and in vivo studies. Their mechanisms of action involve the modulation of key cellular processes associated with cancer development and progression, including the inhibition of cell proliferation, induction of apoptosis, regulation of cell cycle progression, suppression of angiogenesis and metastasis, and modulation of major oncogenic signaling pathways such as PI3K/AKT, MAPK, NF-κB, and EGFR-related cascades. Despite these promising findings, several critical challenges still limit the clinical translation of these compounds. In particular, poor bioavailability due to low solubility, rapid metabolism, and limited absorption remains a major barrier to their therapeutic application. Furthermore, the lack of standardization of plant-derived extracts, including variability in composition depending on source, extraction method, and processing conditions, complicates reproducibility and comparability across studies. Another key limitation is the insufficient clinical validation, as most evidence currently relies on in vitro and preclinical models, with a limited number of well-designed human trials available. Beyond their pharmacological relevance, the recovery of bioactive compounds from plant residues aligns with the principles of the circular economy and sustainable development. The upcycling of agro-industrial by-products not only reduces waste disposal but also creates opportunities for the development of value-added products for the pharmaceutical, nutraceutical, and functional food sectors. Based on the positive effects of these plant-derived compounds, future research should focus on optimizing extraction methods, improving compound stability, enhancing bioavailability, and standardizing plant-derived extracts. In addition, well-designed preclinical and clinical studies will be essential to validate their safety and therapeutic efficacy in humans and to support their potential development as novel anticancer agents.

## Figures and Tables

**Table 1:** Anticancer mechanisms of polyphenols derived from citrus processing waste.

Polyphenol/Compound	Citrus Waste Source	Cancer Type/Model	Molecular Mechanism/Target Pathways	Experimental Model	Reference	Chemical Class/Structural Features
Naringin	Citrus peel, pulp, rag tissues	Breast cancer	Aromatase inhibition leading to reduced estrogen synthesis; decreased tumor cell viability	In vitro (MCF-7, T47D) and in vivo tumor model	[39]	Flavanone glycoside; hydroxyl groups at C5, C7, C4′; glycosylated at C7
Naringenin	Citrus peel and pulp	Breast cancer	Modulation of host immune response leading to suppression of postoperative metastatic growth	In vivo	[47]	Flavanone aglycone; free hydroxyls (C5, C7, C4′); saturated C ring
Naringenin	Citrus peel	Gastric carcinoma	Induction of apoptosis; inhibition of proliferation, migration, and invasion	In vitro	[70]	Same as above
Hesperidin	Citrus peel (orange and lemon peel)	Non-small cell lung cancer	Activation of apoptotic signaling pathways and inhibition of tumor cell growth	In vitro (A549, NCI-H358)	[69]	Flavanone glycoside; methoxy group (C4′), hydroxyl groups; glycosylated
Hesperidin	Citrus peel	Colorectal cancer	Induction of apoptosis and inhibition of cancer cell proliferation	In vivo	[71,72]	Same as above
Ferulic acid (major phenolic acid in lemon peel extract)	Lemon peel	Gastric cancer	Disruption of cellular redox homeostasis; modulation of ROS levels and antioxidant enzymes	In vitro (AGS, MKN-28)	[73]	Hydroxycinnamic acid; phenolic ring + methoxy and hydroxyl substituents; conjugated side chain
Lemon peel polyphenol mixture	Lemon peel	Gastric cancer	Cytotoxic effects through oxidative stress imbalance and altered redox dynamics	In vitro	[73]	Flavonoids and phenolic acids (multiple –OH groups)
Quercetin	Citrus peel	Estrogen-dependent breast cancer	Aromatase inhibition reduces estrogen-mediated tumor growth	In vitro (MCF-7, T47D) and in vivo	[74]	Flavonol; multiple hydroxyl groups (C3, C5, C7, C3′, C4′); C2=C3 double bond
Eriocitrin	Lemon peel and other citrus peels	Hepatocellular carcinoma	Cell cycle arrest; activation of mitochondrial apoptotic pathway; modulation of MAPK signaling	In vitro	[75]	Flavanone glycoside; multiple hydroxyl groups; glycosylated
Eriocitrin	Citrus peel	Tumor angiogenesis models	Inhibition of VEGFR2 phosphorylation; suppression of MAPK-ERK and PI3K/AKT/mTOR signaling pathways	In vitro (endothelial cells)	[76]	Same as above
Eriocitrin	Citrus peel	Tumor invasion and migration processes	Downregulation of MMP-2 and MMP-9, impairing extracellular matrix degradation and endothelial migration	In vitro	[76]	Same as above

Note: Structure–Activity Relationship (SAR) Analysis. The anticancer activity of citrus-derived phenolic compounds is strongly influenced by their structural characteristics. In particular, the number and position of hydroxyl (–OH) groups play a crucial role in determining biological activity, as they enhance antioxidant capacity, facilitate hydrogen bonding with molecular targets, and promote the modulation of key enzymes such as aromatase. The presence of catechol moieties on the B ring further improves radical scavenging and interaction with signaling proteins. Methoxy (–OCH_3_) substitutions, as observed in compounds such as hesperidin and ferulic acid, increase lipophilicity and membrane permeability, although they may reduce the antioxidant potential compared to highly hydroxylated analogs. Glycosylation, typical of compounds like naringin, hesperidin, and eriocitrin, enhances water solubility but generally reduces direct bioactivity, as these molecules often require hydrolysis to their corresponding aglycones to exert stronger intracellular effects. Conversely, aglycone forms such as naringenin exhibit higher cellular uptake and greater biological activity. Additionally, the presence of a C2=C3 double bond in flavonoids (e.g., quercetin) contributes to molecular planarity, improving π–π interactions with proteins and nucleic acids, and thereby enhancing anticancer efficacy. Conjugated side chains, particularly in phenolic acids like ferulic acid, are important for redox modulation, enabling these compounds to regulate reactive oxygen species (ROS) levels and induce oxidative stress in cancer cells. Overall, these structural features collectively determine the ability of phenolic compounds to interact with multiple molecular targets, including signaling pathways (e.g., PI3K/Akt, MAPK), apoptotic regulators, and enzymes involved in tumor progression and metastasis.

**Table 2:** Anticancer mechanisms of polyphenols derived from olive leaf processing waste.

Polyphenol/Compound	Olive Leaves Waste Source	Cancer Type/Model	Molecular Mechanism/Target Pathways	Experimental Model	Reference	Chemical Class/Structural Features
Oleuropein	Olive leaves generated during pruning and olive oil processing	Breast cancer (MCF-7)	Time-dependent inhibition of cell proliferation	In vitro	[89]	Secoiridoid glycoside; ester of hydroxytyrosol and elenolic acid; multiple –OH groups; glycosylated structure
Oleuropein	Olive leaf agro-industrial waste	Triple-negative breast cancer (MDA-MB-231)	Induction of apoptosis via ROS accumulation and modulation of the NF-κB signaling pathway	In vitro	[90]	Same as above
Oleuropein	Olive leaf by-products	Breast cancer (MCF-7, MDA-MB-231)	Reduced cell proliferation, apoptosis induction, inhibition of cell motility and cytoskeletal remodeling	In vitro	[91]	Same as above
Oleuropein	Olive leaf extract and isolated compound	Breast cancer cells	S-phase cell cycle arrest; upregulation of pro-apoptotic genes (BNIP2, BNIP3, BID, BCL10); activation of death receptor signaling (FADD, TNFRSF21); activation of CYCS, CFLAR, GADD45A	In vitro	[92]	Same as above
Oleuropein	Olive leaf waste	Head and neck cancer cells	Apoptosis induction via upregulation of BAX and PTEN; downregulation of CDK2 and CDK4 leading to cell cycle arrest	In vitro	[93]	Same as above
Oleuropein	Olive leaf by-product	Oral squamous cell carcinoma	β-TRCP-mediated ubiquitination and degradation of Mcl-1; inhibition of Akt–GSK3β–Mcl-1 signaling pathway	In vitro and in vivo	[94]	Same as above
Oleuropein	Olive leaf phenolic compound	Prostate cancer (DU145)	DNA damage induction; activation of DNA damage response pathways; modulation of HIF-1α signaling	In vitro	[95]	Same as above
Hydroxytyrosol	Olive leaf agro-industrial waste	Various cancers	Antioxidant activity; reduction in ROS and oxidative stress; chemoprevention through protection against oxidative DNA damage	In vitro	[99]	Simple phenolic alcohol; catechol structure (ortho-dihydroxyl groups)
Hydroxytyrosol	Olive leaf phenolic compound	Multiple cancer models	Modulation of inflammatory pathways; induction of apoptosis and cell cycle arrest; inhibition of the PI3K/AKT and MAPK signaling pathways	In vitro	[103]	Same as above
Hydroxytyrosol	Olive leaf by-products	Acute leukemia (Jurkat, HL-60)	G0/G1 cell cycle arrest and apoptosis via inhibition of the PI3K signaling pathway	In vitro	[104]	Same as above
Hydroxytyrosol	Olive leaf extract	Breast cancer (MCF-7, MDA-MB-231)	Increased expression of pro-apoptotic genes (BAX, caspase-3) and reduced expression of anti-apoptotic BCL-2	In vitro	[105]	Same as above
Hydroxytyrosol	Olive leaves	Colorectal cancer (LS180)	Apoptosis via upregulation of BAX, caspase-3, p53; increased BAX/BCL-2 ratio; suppression of NFE2L2 expression	In vitro	[106]	Same as above

Note: Structure–Activity Relationship (SAR) Analysis. The anticancer activity of olive leaf-derived phenolic compounds is closely associated with their structural features. In particular, the presence of multiple hydroxyl (–OH) groups plays a key role in modulating redox balance, enabling both antioxidant and pro-oxidant effects depending on the cellular context. Hydroxytyrosol, characterized by a catechol structure (ortho-dihydroxyl groups), exhibits strong radical scavenging activity and the ability to chelate metal ions, which contributes to its capacity to regulate oxidative stress and protect against DNA damage while also inducing apoptosis in cancer cells. Oleuropein, a more complex secoiridoid glycoside, combines a phenolic moiety with an elenolic acid ester and a glycosidic linkage, resulting in enhanced stability and bioavailability. Its glycosylated structure influences solubility and cellular uptake, while enzymatic hydrolysis can release the more active hydroxytyrosol moiety. The ester bond and conjugated system in oleuropein contribute to its ability to interact with multiple molecular targets, including signaling proteins involved in apoptosis and cell cycle regulation. Furthermore, the structural complexity of oleuropein enables multitarget activity, including the modulation of the NF-κB, PI3K/AKT, and mitochondrial apoptotic pathways. Overall, simpler phenolics such as hydroxytyrosol tend to exert strong antioxidant and chemopreventive effects, whereas more complex structures like oleuropein exhibit broader mechanisms of action, including direct cytotoxicity, gene regulation, and interference with tumor progression pathways.

**Table 3:** Anticancer mechanisms of polyphenols derived from date palm residues.

Polyphenol/Compound	Date Palm Residues Waste Source	Cancer Type/Model	Molecular Mechanism/Target Pathways	Experimental Model	Reference	Chemical Class/Structural Features
Date seed extract (polyphenol-rich)	Date palm seeds/pits (processing by-product)	Hepatic, colorectal, and breast cancer cells	Antioxidant and metal-chelating activity; inhibition of acetylcholinesterase, α-amylase, and tyrosinase; apoptosis induction with BCL-2 downregulation and p53 upregulation	In vitro	[112]	Complex mixture of phenolic acids and flavonoids; multiple –OH groups; high redox potential
Date pit extract	Date palm pits from Emirati cultivars	Triple-negative breast cancer (MDA-MB-231)	Cytotoxicity mediated through EGFR/ERK/FAK signaling, Src family kinases, and modulation of calcium signaling pathways	In vitro	[113]	Mixed polyphenols; flavonoids and phenolic acids
Date pit extract	Date palm pits	Pancreatic cancer cell lines	Antiproliferative and pro-apoptotic activity	In vitro	[114]	Same as above
Date seed extract	Date palm seeds (Khalas cultivar)	Prostate and pancreatic cancer cells	Apoptosis induction with G2/M cell cycle arrest and upregulation of caspase-3 and caspase-9	In vitro	[115]	Mixed phenolics; flavonoid-rich
Date palm leaf extract	Date palm leaves (Khalas cultivar)	Prostate and pancreatic cancer cells	Cytotoxic and antiproliferative effects associated with high phenolic and flavonoid content and enhanced antioxidant activity	In vitro	[115]	Polyphenol and flavonoid mixture; hydroxylated compounds
Date palm leaf extract	Leaves from four cultivars	Breast cancer (MDA-MB-231) and glioblastoma (U87)	Inhibition of tumor cell adhesion and migration through interference with αvβ3 and α5β1 integrin receptors	In vitro	[116]	Same as above
Syringic acid	Date palm residues (seeds and flesh)	Colorectal cancer (DMH-induced)	Inhibition of proliferation; ROS-mediated apoptosis and DNA damage; modulation of the Nrf2, NF-κB, and PI3K/AKT signaling pathways	In vivo (rat model)	[117]	Phenolic acid; methoxy (–OCH_3_) and hydroxyl substitutions on aromatic ring
Quercetin	Date palm seeds and flesh	Prostate cancer	Apoptosis induction; inhibition of proliferation via androgen receptor (AR) and PI3K/AKT pathway suppression	In vivo	[118]	Flavonol; polyhydroxylated (C3, C5, C7, C3′, C4′); planar structure (C2=C3 double bond)
Quercetin	Date palm residues	Prostate cancer	Anti-angiogenic activity via thrombospondin-1 (TSP-1) modulation	In vivo	[119]	Same as above
Quercetin	Date palm seeds and flesh	Breast cancer	Apoptosis induction; inhibition of metastasis and glycolysis; suppression of VEGF, PKM2, and p-AKT/AKT signaling	In vivo	[121]	Same as above
Quercetin	Date palm residues	Various cancer models	Cell cycle arrest and inhibition of the AKT/mTOR pathway; anti-angiogenic effects via calcineurin/NFAT signaling inhibition	In vitro/In vivo	[122]	Same as above
Quercetin	Date palm residues	Colorectal cancer	Reduced metastasis and induction of apoptosis	In vitro/In vivo	[123]	Same as above
Quercetin	Date palm residues	Hepatocellular carcinoma	Tumor growth inhibition	In vivo	[124]	Same as above
Quercetin	Date palm residues	Triple-negative breast cancer	Suppression of stress-induced proliferation and migration via inhibition of β2-adrenergic receptor–ERK1/2 signaling	In vitro/In vivo	[125]	Same as above
Ferulic acid	Date palm residues (flesh and seeds)	Multiple cancer models	Inhibition of PI3K/AKT and JAK2/STAT6 signaling pathways leading to apoptosis and tumor growth suppression	In vitro/In vivo	[127]	Hydroxycinnamic acid; conjugated side chain; methoxy + hydroxyl groups
Gallic acid	Date palm residues	Human embryonic carcinoma (NTERA-2, NCCIT)	G0/G1 cell cycle arrest, apoptosis induction, suppression of EGFR/JAK2/STAT5, downregulation of SOX2, NANOG, OCT4	In vitro	[128]	Simple phenolic acid; trihydroxylated benzene ring
Caffeic acid	Date palm residues	Malignant pleural mesothelioma	Inhibition of ERK1/2 and AKT signaling; G2/M arrest via p53-p21/p27 axis; mitochondrial apoptosis with increased Bax/Bcl-2 ratio and caspase-3 activation	In vitro	[129]	Hydroxycinnamic acid; catechol structure + conjugated double bond

Note: Structure–Activity Relationship (SAR) Analysis. The anticancer activity of phenolic compounds derived from date palm residues is strongly governed by their structural diversity, ranging from simple phenolic acids to complex flavonoids and polyphenol-rich mixtures. A key determinant of activity is the number and arrangement of hydroxyl (–OH) groups, which enhance redox properties, allowing these compounds to act as both antioxidants and pro-oxidants in cancer cells. Polyhydroxylated structures such as quercetin exhibit high biological activity due to their ability to form hydrogen bonds with proteins, modulate enzyme activity, and interact with key signaling pathways, including PI3K/AKT and mTOR. The presence of a C2=C3 double bond and a planar flavonol structure further enhances binding affinity to molecular targets through π–π interactions. Phenolic acids such as caffeic and ferulic acid benefit from conjugated side chains, which stabilize radical intermediates and facilitate the modulation of oxidative stress and signaling cascades. Catechol moieties (as in caffeic acid) significantly improve ROS-scavenging capacity and metal-chelating ability, while methoxy substitutions (as in syringic and ferulic acid) increase lipophilicity and influence membrane permeability and bioavailability. Simple phenolics like gallic acid, with multiple hydroxyl groups, are particularly effective in inducing apoptosis and cell cycle arrest via oxidative stress-mediated mechanisms. Additionally, complex mixtures present in date seed and leaf extracts exhibit synergistic effects due to the coexistence of multiple bioactive phenols, enhancing multitarget interactions such as the inhibition of kinase signaling, modulation of apoptosis-related proteins, and interference with tumor cell adhesion and migration. Overall, these structural features collectively determine the ability of date palm phenolics to regulate cancer-related pathways and exert potent anticancer effects.

**Table 4:** Anticancer mechanisms of polyphenols derived from tea and coffee by-products.

Polyphenol/Compound	Tea and Coffee By-Products Waste Source	Cancer Type/Model	Molecular Mechanism/Target Pathways	Experimental Model	Reference	Chemical Class/Structural Features
Chlorogenic acid	Coffee by-products (coffee grounds, husk, pulp, silverskin)	General cancer models	Induces cell cycle arrest, promotes apoptosis, and inhibits tumor cell proliferation; modulation of immune-related genes and induction of DNA damage	In vitro	[139]	Hydroxycinnamic acid ester (caffeic acid + quinic acid); catechol moiety; conjugated system
Chlorogenic acid (coffee extracts)	Coffee by-products/green and roasted coffee extracts	Hepatocellular carcinoma (HepG2)	Dose-dependent inhibition of cell viability and proliferation; apoptosis-related mechanisms with minimal involvement of the Wnt/β-catenin pathway	In vitro	[140]	Same as above
Coffee bioactive compounds (chlorogenic acid-rich extracts)	Coffee by-products	Colorectal cancer models	Cell cycle arrest at G0/G1 phase, reduced proliferation, and induction of apoptosis	In vitro/In vivo	[141]	Mixture of phenolic acids; high catechol content
Coffee roasted powder extract	Coffee grounds	Human colon cancer (Caco-2)	Upregulation of miR-30c and miR-96 and suppression of KRAS proto-oncogene, leading to inhibition of tumor cell growth	In vitro	[142]	Complex mixture of phenolics, Maillard products, and flavonoids
Epigallocatechin gallate (EGCG)	Tea waste (Camellia sinensis residues)	Lung cancer (A549, HCC827, H1975)	Inhibition of EGFR signaling; altered phosphorylation of mTOR, p38 MAPK, and STAT3; apoptosis induction and reduced migration through modulation of vinculin and metavinculin	In vitro	[147]	Flavan-3-ol (catechin); gallate ester; multiple hydroxyl groups; highly polyhydroxylated
Epigallocatechin gallate (EGCG)	Tea waste/green tea residues	Bladder cancer (5637, T24)	Inhibition of proliferation and induction of apoptosis via caspase-9, caspase-3, BAX activation; modulation of autophagy-related proteins LC3B-II, Beclin; involvement of PI3K/AKT and ATG5 pathways	In vitro	[148]	Same as above
EGCG and green tea extract	Tea by-products	Breast cancer (MCF-7 spheroid model)	Suppression of spheroid formation and cell migration; enhanced anticancer activity due to synergistic phytochemical interactions	In vitro (3D model)	[149]	Polyphenol mixture rich in catechins (EGCG dominant)
EGCG and green tea extract	Tea by-products	Melanoma	Induction of apoptosis, inhibition of proliferation, anti-angiogenic effects, and suppression of TNF-α-induced VEGF and IL-8 secretion	In vitro	[150]	Same as above

Note: Structure–Activity Relationship (SAR) Analysis. The anticancer activity of phenolic compounds derived from tea and coffee by-products is largely determined by their structural features, particularly the presence of catechol groups, conjugated systems, and the degree of hydroxylation. Chlorogenic acid, a hydroxycinnamic acid ester composed of caffeic and quinic acid, contains a catechol moiety that enhances its antioxidant and metal-chelating properties, enabling the modulation of oxidative stress and induction of apoptosis in cancer cells. The conjugated double bond within its structure further stabilizes radical intermediates and facilitates interaction with cellular targets involved in proliferation and immune regulation. Epigallocatechin gallate (EGCG), a highly polyhydroxylated flavan-3-ol with a gallate ester group, exhibits strong biological activity due to its multiple hydroxyl groups, which promote hydrogen bonding with proteins and enzymes, and its planar structure, which enhances binding affinity to signaling molecules such as EGFR and kinases involved in the PI3K/AKT and MAPK pathways. The gallate moiety is particularly important for increasing anticancer potency by improving interactions with lipid membranes and protein targets. Additionally, complex mixtures such as coffee extracts and green tea residues exhibit synergistic effects due to the coexistence of multiple phenolic compounds, leading to multitarget mechanisms including the modulation of gene expression (e.g., microRNAs), inhibition of oncogenes such as KRAS, and suppression of angiogenesis-related factors. Overall, structural elements such as hydroxyl group density, esterification, and conjugation play a central role in determining the ability of these compounds to regulate key cancer-related pathways and exert antiproliferative and pro-apoptotic effects.

## Data Availability

No new data were created or analyzed in this study. Data sharing is not applicable to this article.
